# Caspofungin enhances the potency of rifampin against Gram-negative bacteria

**DOI:** 10.3389/fmicb.2024.1447485

**Published:** 2024-08-15

**Authors:** Haotian Li, Xiaojing Zhu, Xing Zhang, Changjiang Dong

**Affiliations:** ^1^School of Pharmaceutical Sciences, Wuhan University, Wuhan, China; ^2^Key Laboratory of Combinatorial Biosynthesis and Drug Discovery, Ministry of Education and School of Pharmaceutical Sciences, Wuhan University, Wuhan, China

**Keywords:** antimicrobial resistance, Gram-negative bacteria, antibiotic adjuvant, bacterial envelope, PgaC

## Abstract

**Introduction:**

Developing antibiotic adjuvants is an effective strategy to combat antimicrobial resistance (AMR). The envelope of Gram-negative bacteria (GNB) is a barrier to prevent the entry of antibiotics, making it an attractive target for novel antibiotic and adjuvant development.

**Methods and Results:**

In this study, we identified Caspofungin acetate (CAS) as an antibiotic adjuvant against GNB in the repurposing screen of 3,158 FDA-approved drugs. Checkerboard assays suggested that CAS could enhance the antimicrobial activity of rifampin or colistin against various GNB strains *in vitro*, Moreover, *Galleria mellonella* larvae infection model also indicated that CAS significantly potentiated the efficacy of rifampin against multidrug-resistant *Escherichia coli* 72 strain *in vivo*. Most importantly, resistance development assay showed that CAS was less susceptible to accelerating the resistance development of drug-sensitive strain *E. coli* MG1655. Functional studies and RNA-seq analysis confirmed that the mechanisms by which CAS enhanced the antimicrobial activities of antibiotics were involved in permeabilizing the bacterial cell envelope, disrupting proton motive force and inhibiting bacterial biofilm formation. Additionally, it has been found that PgaC is the CAS target and enzymatic assay has confirmed the inhibition activity.

**Discussion:**

Our results illustrate the feasibility of CAS as an antibiotic adjuvant against GNB, which is an alternative strategy of anti-infection.

## 1 Introduction

Antimicrobial resistance (AMR) has posed a huge threat to human and animal health worldwide (Croft et al., 2007; Ferri et al., 2017; Samreen et al., 2021). The statistics of the World Health Organization (WHO) in 2019 showed that AMR caused at least 700,000 deaths every year (Mancuso et al., 2021). The six major pathogenic bacteria that lead to deaths include “ESKAPE” (*Enterococcus faecium*, *Staphylococcus aureus*, *Klebsiella pneumoniae*, *Acinetobacter baumannii*, *Pseudomonas aeruginosa*, and *Enterobacter* species) (Collaborators A.R, 2022), most of which are Gram-negative bacteria (GNB). It is reported that we are facing the threat of untreatable multidrug-resistant Gram-negative pathogenic bacteria infections (Karaiskos and Giamarellou, 2014).

Although great efforts have been made to develop novel strategies to unravel bacterial resistance, which include the development of antibiotic alternatives, such as vaccines, probiotics, and phage therapy (Allen et al., 2014; Hoelzer et al., 2018; Browne et al., 2020), the AMR rate is still sharply growing. Thus, novel antimicrobial strategies are urgently needed to combat AMR.

Considering the difficulty in developing novel antimicrobials, the discovery of antibiotic adjuvants is a meaningful point for combating AMR (Kumar et al., 2023). Antibiotic adjuvants are a class of compounds that commonly show little or no antimicrobial activity themselves but can block resistance and promote antibiotic activity by targeting efflux pumps, modifying enzymes, bacterial cell permeability, or host defense systems (Douafer et al., 2019). They have been extensively used in clinical therapy; for example, clavulanic acid and sulbactam are used to enhance the antimicrobial activities of β-lactam antibiotics (Reading and Cole, 1977; Kanra, 2002). It is established that antibiotic adjuvants can greatly extend the clinical existing antibiotic lifespan (González-Bello, 2017).

GNB possess intrinsic antibiotic resistance, which can be mainly attributed to their envelope barrier (Maher and Hassan, 2023), especially the asymmetrical outer membrane (OM) (Delcour, 2009). Bacterial OM biogenesis is involved in many protein complex machineries, including BamABCDE (OM proteins folded machinery), LolCDE (OM lipoprotein transporter), LptB_2_FGC [lipopolysaccharide (LPS) transporter] (Choi and Lee, 2019), all of which are the promising targets for antimicrobials (Naclerio and Sintim, 2020). There are some compounds targeting these complexes that have been identified as antimicrobials; for example, a BamA inhibitor, darobactin, was found to have great antimicrobial potency (Imai et al., 2019), and zosurabalpin was also identified as antimicrobial by targeting the LPS transporter LptB_2_FGC (Zampaloni et al., 2024). In addition, several compounds have also been screened out to disrupt the integrity of bacterial OM, recovering the efficacy of existing antibiotics against AMR strains, such as SLAP-S25 and LL-17 (Shurko et al., 2018; Song et al., 2020; Yang et al., 2022). Hence, bacterial OM is also a promising target for antibiotic adjuvants.

Hydrophobic antibiotics have difficulty entering the cytoplasm of GNB (Savage, 2001), resulting in a significantly limited variety of antibiotics available for treating GNB infections. Rifampin is a classical hydrophobic antibiotic for the treatment of infections caused by not only *Tuberculosis mycobacteria* but also other bacterial pathogens such as *S. aureus* (Lee et al., 2017). Notably, rifampin was sometimes used for the treatment of GNB pathogens in clinics; for example, the combination of carbapenem and rifampin successfully cured the infection of hypervirulent *K. pneumoniae* bacteremia (Lin et al., 2021). Many previous studies also showed that the antimicrobial activities of rifampin against GNB pathogens could be enhanced by several bacterial membrane disruptors, such as D-LANA-14 and ACP-1 (Gly) (Barman et al., 2019; Konai and Haldar, 2020). Taken together, the synergy of bacterial membrane disruptors and hydrophobic antibiotics is a promising antimicrobial strategy to combat GNB pathogens.

In this study, we established a high-throughput method of screening antibiotic adjuvants targeting bacterial OM and performed FDA-approved drug repurposing. Casoufungin acetate (CAS) was identified as a potential antibiotic adjuvant to enhance the antimicrobial activities of rifampin or colistin against GNB strains *in vitro* or *in vivo*. Our data also showed that the synergistic effects of CAS in combination with antibiotics were driven by destructing the bacterial envelope, dissipating proton motive force (PMF), and inhibiting biofilm formation. In particular, we identified that PgaC might be the CAS target, and an enzymatic assay showed that the CAS inhibits PgaC activity. In summary, this study provides an antibiotic adjuvant candidate that is worth further evaluating and exploring.

## 2 Materials and methods

### 2.1 Bacterial strains and drug library

Bacterial strains and plasmids used in this study are listed in Supplementary Table 1. All strains were cultured in a lysogeny broth (LB) medium. The FDA-approved drug library (3,158 compounds) was purchased from Targetmol, and the detailed information is listed in Supplementary Table 2. The library was supplied in 96-well plates of 10 mM stocks in dimethyl sulfoxide (DMSO) and stored at −80°C.

### 2.2 High-throughput screening for antibiotics against *Escherichia coli* MG1655

To screen for antibiotic adjuvants against GNB, the mid-log phase cells of *E. coli* MG1655 were diluted in LB medium with 2 μg/mL rifampin in 96-well plates containing library drugs or polymyxin B nonapeptide (PMBN, positive control) or dimethyl sulfoxide (DMSO, negative control). After 8 h of static culture at 37°C, the optical density at 600 nm (OD_600_) was measured using a microplate reader. Percentage inhibition was calculated as (OD_N_ – OD_X_)/(OD_N_ – OD_P_) × 100%, where OD_X_ is the OD_600_ value for a test treated with drug X, and OD_P_ and OD_N_ are the OD_600_ values for the positive and negative control, respectively.

### 2.3 Checkerboard assay

Checkerboard assays were performed to evaluate the synergistic effect of CAS in combination with antibiotics as previously described (Cai et al., 2023). Briefly, antibiotics and CAS were diluted two times with MHB medium (with or without Mg^2+^ or EDTA) in a 96-well plate, respectively, to form an 8 × 8 medium, and an equal volume (200 μL/well) of bacterial suspension with the drug was cultured. A microplate reader was used to measure the OD_600_ value after 18 h of culture at 37°C (the OD_600_ value over 0.1 indicates bacterial growth). The fractional inhibitory concentration index (FICI) was calculated using the following formula: FICI = MIC_ab_/MIC_a_ + MIC_ba_/MIC_b_. MICa and MIC_b_ are the corresponding MIC values of compounds A and B alone, respectively; MIC_ab_ is the MIC value of compound A combined with compound B; and MIC_ba_ is the MIC value of compound B combined with compound A. Indicative of synergy is FICI ≤0.5.

### 2.4 Time-kill curve against *Escherichia coli*

The bactericidal curves of CAS against drug-sensitive and resistant *E. coli* strains were determined, respectively. The 10^6^ CFU of *E. coli* MG1655 and 72 strains were washed and resuspended in an MHB medium, then treated with CAS and rifampin alone or in combination, and a control group without any treatment. At each time point (0, 4, 8, and 24 h), 100 μL of the bacterial solution was continuously diluted 10-fold in physiological saline. Subsequently, the suspension was then plated onto LB agar plates and cultured for an overnight period at 37°C for bacterial count.

### 2.5 Outer membrane permeability assay

The fluorescent probe N-phenyl-1-naphthylamine (NPN) was used to evaluate the outer membrane integrity of *E. coli* treated by CAS or combined with rifampin as described previously (Song et al., 2020). Briefly, the mid-phase cells of *E. coli* were washed and suspended with 5 mM of HEPES (pH 7.0 + 5 mM of glucose). Then, the samples were standardized to an OD_600_ value of 0.5, and the dye NPN was added to a final concentration of 10 μM. After incubation at 37°C for 30 min, 190 μL of probe-labeled bacterial cells were added to a 96-well plate, and then CAS or rifampin was added. After incubation for 30 min, the fluorescence intensity was measured on a microplate reader with the excitation wavelength at 350 nm and the emission wavelength at 420 nm.

### 2.6 Membrane integrity assay

Fluorescent probe PI was used to assess the inner membrane integrity of *E. coli* treated by CAS or combined with rifampin as described previously (Song et al., 2020). Briefly, the mid-phase cells of *E. coli* were washed and suspended in a phosphate-buffered solution (PBS). Then, the samples were standardized to an OD_600_ value of 0.5, followed by the addition of 10 nM of propidium iodide (PI) in the presence of CAS or combined with rifampin. After incubation for 30 min, the samples were measured on a microplate reader with the excitation wavelength at 535 nm and the emission wavelength at 615 nm.

### 2.7 Extracellular β-galactosidase determination

The mid-phase cells of *E. coli* were washed and resuspended to obtain an OD_600_ value of 0.5 with PBS buffer. Then, the samples were treated with CAS or combined with rifampin for 1 h at 37°C and centrifuged at 12,000 rpm for another 10 min at 4°C. The supernatants were collected to determine the activity of β-galactosidase. Briefly, 190 μL of the supernatants were added to each well in a 96-well microplate, followed by the addition of a final concentration of 3 mM of 2-nitrophenyl-β-d-galactopyranoside (ONPG, a substrate of β-galactosidase). After incubation at 37°C for 30 min, the absorbance at 420 nm was measured using a microplate reader.

### 2.8 Measurement of membrane potential

The mid-phase *E. coli* cells were washed and resuspended to obtain an OD_600_ value of 0.5 with 5 mM of HEPES (pH 7.0 + 5 mM of glucose). The fluorescent probe 3,3′-Dipropylthiadicarbocyanine iodide (DiSC_3_(5)) (a final concentration of 0.5 μM) was added and incubated for 30 min. The probed cells of *E. coli* were mixed with a variety of concentrations of CAS (8, 16, 32 μg/mL), then measured using an excitation wavelength at 622 nm, and an emission wavelength at 670 nm with a microplate reader.

### 2.9 Measurement of bacterial inner pH

The mid-phase *E. coli* cells were washed and resuspended to obtain an OD_600_ value of 0.5 with 5 mM of HEPES (pH 7.0 + 5 mM of glucose) and the final concentration of 2 μM pH-sensitive fluorescent probe 2′,7′-bis-(2 carboxyethyl)-5-(and-6)-carboxyfluorescein, acetoxymethyl ester (BCECF-AM) was added. After incubation at 37°C for 30 min, the samples were treated with a variety of concentrations of CAS (8, 16, 32 μg/mL) and incubated at 37°C for 30 min. The fluorescence intensity was immediately monitored with the excitation wavelength of 488 nm and emission wavelength of 535 nm.

### 2.10 Biofilm formation assay

Biofilm formation of *E. coli* was measured as described previously with minor modifications (Wang et al., 2004). Briefly, the mid-log phase cells of *E. coli* were transformed into LB medium containing 0.2% glucose and CAS or combined with rifampin, then incubated at 26°C for 48 h. The planktonic bacteria were removed, and the biofilm was washed with PBS. Subsequently, the samples were fixed with methanol for 15 min, stained with 1% crystal violet for 10 min, and then washed with PBS. After adding 33% glacial acetic acid to dissolve the dye, the absorbance at 595 nm (OD_595_) was recorded with a microplate reader. Biofilm formation was also visualized by aliquoting 1 mL of diluted culture into 1.5 mL polystyrene microtubes and incubating statically at 26°C for 48 h. Biofilms were then stained by the addition of 200 μL of crystal violet and incubated for 15 min, washed three times with PBS buffer, and photographed.

### 2.11 RNA-seq analysis

To analyze the transcriptome changes of *E. coli* MG1655 after CAS treatment, RNA-seq was conducted by MAGIGENE, Guangzhou. Briefly, RNA samples were extracted from the mid-log phase cells of the strains using the Trizol method. The quality control of RNA samples was checked by agarose gel electrophoresis, Thermo NanoDrop One, and Agilent 4,200 Tape Station. The ribosomal RNA was removed from the samples, and the library was constructed using the NEBNext^®^ Ultra II™ Directional RNA Library Prep Kit. The library was sequenced using the Illumina HiSeq/MiSeq platform. Quality control of raw reads was performed, and clean reads were mapped onto the *E. coli* MG1655 genome. The gene expression levels were analyzed using the feature counts (Dillies et al., 2013) and DEseq2 (Love et al., 2014). The DEGs were identified by setting the threshold |log2 (foldchange) | ≥1 and a *p*-value of ≤0.05. The enrichment based on the KEGG pathway database was analyzed.^1^ To validate the DEGs, quantitative RT-PCR was performed using primers listed in Supplementary Table 3 (QuantStudio 6 Flex, Micromeritics, Shanghai, China). The relative levels of target gene expression were normalized with the *gapdh* gene using the 2^−∆∆Ct^ method.

### 2.12 Swarming assay

The effect of CAS on the swarming motility of *E. coli* was investigated as described previously with minor modifications (She et al., 2022). Briefly, the medium for the motility assay was LB containing 0.5% agar and CAS (a final concentration at 64 μg/mL). Then, 2 μL volume of *E. coli* MG1655, 72 strains cultured at an OD_600_ of 0.5 were placed in the center of each plate, respectively, and allowed to stay for 30 min. The plates were placed in a 37°C incubator for 24 h.

### 2.13 Construction of the expression plasmid of PgaCD of *Escherichia coli*

The plasmid pTrc99a-*pgaC*^V227L^*pgaD*^N75D/K76E^ encodes the variant PgaCD of *E. coli* with a C-terminal strep tag in *E. coli* expression system, of which the enzymatic activity is independent of the regulation of c-di-GMP (Steiner et al., 2013). The coding sequence of the variant *pgaCD* was amplified by overlap extension PCR and subsequently cloned into the pTrc99a vector by homologous recombination. All the primers used in this study are listed in Supplementary Table 3.

### 2.14 Protein expression and purification

The strep-tagged recombinant proteins were expressed and purified as described previously. *E. coli* C43 (DE3) containing the plasmid pTrc99a-*pgaC*^V227L^*pgaD*^N75D/K76E^ was grown to the mid-log phase, and protein expression was induced by the addition of 0.2 mM isopropyl-β-D-thiogalactopyranoside (IPTG), followed by incubation at 18°C for 16 h. The cells were harvested and lysed by high-pressure crushing. The cell lysate was subjected to centrifugation at 12,000 rpm for 20 min at 4°C to remove the unbroken cells and cell debris. The supernatant was then subjected to ultracentrifugation at 36,300 rpm at 4°C to obtain the membrane fractions. The membrane fractions were further solubilized in Buffer A (50 mM HEPES pH 8.0, 300 mM NaCl, 5% v/v glycerol, 1 mM TCEP) supplemented with 1% w/v DDM/0.1% w/v CHS for 60 min at 4°C. Insoluble material was removed by centrifugation at 16, 000 g for 30 min at 4°C. The supernatant was applied to a gravity flow chromatography column packed with 2 mL of Streptactin Beads 4FF (Smart-Lifescience) and incubated at 4°C for 30 min. The beads were washed with 40 mL of Buffer A supplemented with 0.1% w/v DDM/0.01% w/v CHS, and eluted with 4 mL of buffer A supplemented with 0.1% w/v DDM/0.01% w/v CHS and 2.5 μM d-Desthiobiotin. Protein was concentrated by ultrafiltration and verified using the NanoDrop100 Spectrophotometer (Thermo Fisher Scientific).

### 2.15 PgaCD enzymatic activity assay

PgaCD glycosyltransferase activity assay was performed as described previously with minor modifications (Steiner et al., 2013). Briefly, 50 μL reaction mixtures containing PgaCD complex (0.32 mg/mL) in glycosyltransferase activity buffer (50 mM HEPES pH 8.0, 300 mM NaCl, 5% v/v glycerol, 1 mM TCEP, 5 mM MgCl_2_) and a variety of concentrations of CAS (final concentrations of 0, 16, and 32 μg/mL) were incubated for 18 h at 37°C with or without 2 mM UDP-GlcNAc. The samples were centrifuged at 12,000 rpm at 4°C for 5 min. The supernatants were added with shrimp alkaline phosphatase and incubated for 30 min at room temperature, followed by incubation at 65°C for 5 min. Phosphate content (indirect measure for UDP) was determined spectrophotometrically at 630 nm using the color reagent containing molybdate and malachite green.

### 2.16 Resistance development study

The resistance development study was performed as described previously with minor modifications (Konai and Haldar, 2015). Briefly, *E. coli* MG1655 overnight cultures were diluted 1:100 in LB broth containing 0.5 × MIC of rifampin or combined with 8 μg/mL CAS. After 12 h of incubation at 37°C, the bacterial culture was diluted 1:100 in fresh LB broth containing 0.5 × MIC of rifampin or combined with 8 μg/mL CAS to continue the next generation. In every three passages, the MIC of the cultures was measured. The process was repeated for 15 passages.

### 2.17 *Galleria mellonella* infection model

The *G. mellonella* larvae model was used to evaluate the virulence of *E. coli* as described previously (Song et al., 2020). A total of 40 *G. mellonella* larvae were randomly divided into four groups (10 per group), which were injected with 2.25 × 10^5^ CFU of *E. coli* 72, or the same volume of saline, respectively, via the leaf posterior proleg of *G. mellonalla* larvae, followed by CAS (64 mg/kg) and rifampin (16 mg/kg) alone or in combination administrations via the right posterior proleg. The survival of *G. mellonella* larvae was recorded at 6 h intervals for 48 h.

### 2.18 Statistical analysis

The data were analyzed using a two-tailed Student’s *t*-test or log-rank test in GraphPad Prism 7 software, with a *p*-value of <0.05 considered to be statistically significant.

## 3 Results

### 3.1 Primary screening of FDA-approved drug library

**Figure 1:**
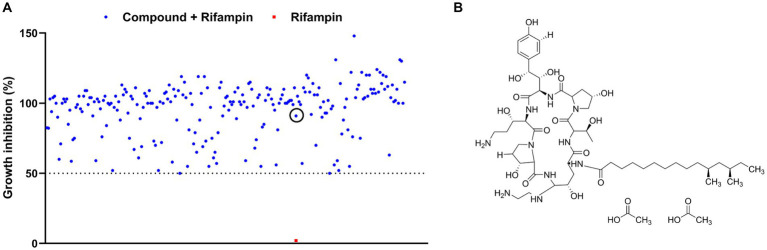
Screening for antibiotic adjuvants. **(A)** The scatter plot of the growth inhibition data of rifampin at 2 μg/mL alone or combined with 235 FDA-approved drugs against *E. coli* MG1655, respectively. The red solid square and blue solid circle in the black circle represented the inhibition data of rifampin alone and in synergy with CAS against *E. coli* MG1655, respectively; **(B)** The structure of CAS.

An FDA-approved library containing 3,158 compounds was subjected to testing for synergistic antimicrobial activity with rifampin (at a concentration without bioactivity) against the *E. coli* MG1655 strain. As shown in Figure 1A, 235 hits in combination with rifampin exhibited a growth inhibition of over 50% (Supplementary Table 4). Subsequently, the individual efficacies of all the hits against the *E. coli* MG1655 strain were measured, respectively. Of these hits, the growth inhibitions of 221 hits alone against *E. coli* MG1655 were higher than 50% (Supplementary Table 4), but the 221 hits belong to known antibiotics and identified non-antibiotic compounds with antimicrobial activity. Of the other 14 hits, CAS, an antifungal agent with weak antimicrobial efficacy (Figure 1B), significantly enhanced the potency of rifampin, which hinted that CAS could potentiate the antimicrobial activity of rifampin against *E. coli* MG1655.

### 3.2 CAS potentiates the efficacy of rifampin and colistin against Gram-negative bacteria *in vitro*

**Figure 2:**
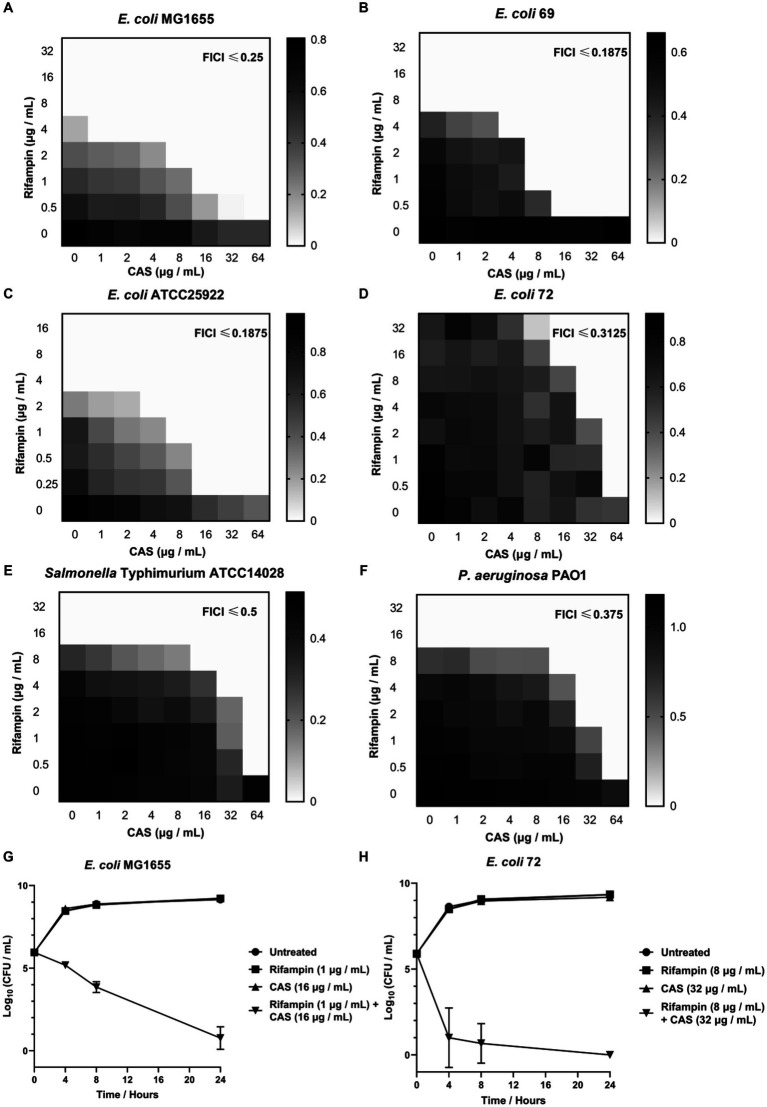
The synergistic effect of CAS with rifampin against *E. coli*. The synergistic effect of CAS with rifampin against **(A)** *E. coli* MG1655, **(B)** *E. coli* 69, **(C)** *E. coli* ATCC25922, **(D)** *E. coli* 72, **(E)** *S. typhimurium* ATCC14028, and **(F)** *P. aeruginosa* PAO1, respectively. The grayscale values of the 8 × 8 checkerboard represent the OD_600_ values, and an FIC index of <0.5 is used to define synergy. **(G)** 1 × 10^6^ CFU *E. coli* MG1655 was incubated with CAS (16 μg/mL), rifampin (1 μg/mL), or synergy for 24 h, respectively. Next, the samples were counted at 4, 8, and 24 h. **(H)** 1 × 10^6^ CFU multidrug-resistant strain *E. coli* 72 was incubated with CAS (32 μg/mL), rifampin (8 μg/mL), or synergy for 24 h, respectively. Next, the samples were counted at 4, 8, and 24 h.

To determine the degree of synergy of CAS with rifampin or colistin against GNB, we performed checkerboard assays to determine FICI using several GNB strains. It was shown that CAS solely displayed a weak antibacterial activity against all tested *E. coli* strains, with the minimum inhibitory concentration (MIC) higher than or equal to 128 μg/mL, but it could decrease the MIC values of rifampin against *E. coli* MG1655 (from 8 μg/mL to 0.5 μg/mL) (Figure 2A), *E. coli* 69 (from 8 μg/mL to 0.5 μg/mL) (Figure 2B), *E. coli* ATCC25922 (from 4 μg/mL to 0.25 μg/mL) (Figure 2C), and multidrug-resistant strain *E. coli* 72 (from 64 μg/mL to 0.5 μg/mL) (Figure 2D), and enhance the bioactivity of colistin against these strains except *E. coli* 72 (Supplementary Table 5). Additionally, CAS was also found to decrease the MIC values of rifampin against *Salmonella Typhimurium* ATCC14028 (from 16 μg/mL to 0.5 μg/mL) (Figure 2E) as well as *P. aeruginosa* PAO1 (from 16 μg/mL to 0.5 μg/mL) (Figure 2F) and act synergistically with colistin against these two strains (Supplementary Table 5). Collectively, CAS could be confirmed as a potential adjuvant for rifampin or colistin against Gram-negative pathogens. To further evaluate the synergistic antimicrobial effect of CAS in combination with rifampin against drug-sensitive and resistant *E. coli* strains, the time-kill tests were performed for the tested strains, including *E. coli* MG1655 and *E. coli* 72. The results showed that CAS and rifampin at a specific dose alone exhibited no antimicrobial efficacy against these two strains, while CAS in combination with rifampin greatly killed these two strains (Figures 2G,H). Taken together, CAS indeed enhances the efficacy of rifampin or colistin against drug-sensitive or resistant GNB strains, which implies that the mode of action of CAS might not be limited to targeting the resistant mechanism.

### 3.3 CAS impaired bacterial envelope

**Figure 3:**
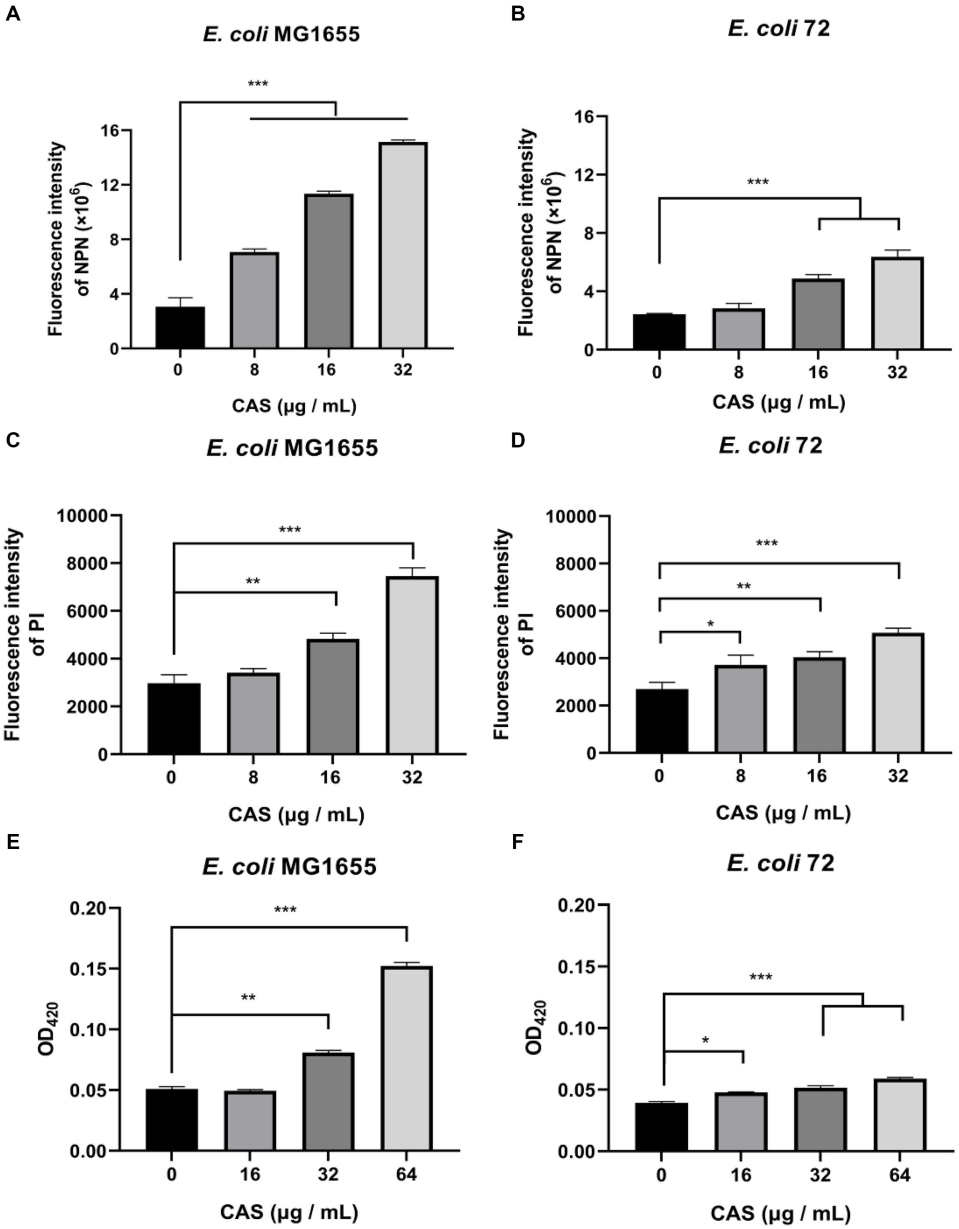
CAS impairs the envelope integrity of *E. coli*. The dye NPN (a final concentration of 10 μM) probed **(A)** *E. coli* MG1655 and **(B)** *E. coli* 72 were incubated with CAS (a final concentration of 8, 16, and 32 μg/mL) for 30 min, respectively. Next, fluorescence was measured on a microplate reader with the excitation wavelength at 350 nm and the emission wavelength at 420 nm. The PI (a final concentration of 10 nM) was added to the cells of **(C)** *E. coli* MG1655 and **(D)** *E. coli* 72 in the presence of CAS (a final concentration of 8, 16, and 32 μg/mL), respectively. After incubation for 30 min, fluorescence was measured with an excitation wavelength of 535 nm and an emission wavelength of 615 nm. The cells of **(E)** *E. coli* MG1655 and **(F)** *E. coli* 72 treated with CAS (a final concentration of 16, 32, and 64 μg/mL) were centrifuged, and the supernatants were incubated with a final concentration of 3 mM ONPG for 30 min. The absorbance at 420 nm was measured using a microplate reader. The data were analyzed by a two-tailed Student’s *t*-test in GraphPad Prism 7 software, with a value of *p < 0.05, **p < 0.01, and ***p < 0.001. The experiments were carried out two times independently under the same conditions.

The envelope of GNB is composed of an asymmetric OM, a thin peptidoglycan, and a cytoplasmic or inner membrane (IM) (Saha et al., 2021), which is a protective barrier. The bacterial envelope disruptors can potentiate the efficacy of hydrophobic antibiotics by destroying the envelope. To explore the antimicrobial mechanisms of the combination of CAS and rifampin, we first evaluated the OM and IM integrities of cells of *E. coli* treated with CAS. The results showed that the dose-dependent increases in fluorescence intensities of dye NPN-labeled cells of *E. coli* MG1655 and multidrug-resistant *E. coli* 72 treated with CAS alone were observed, respectively (Figures 3A,B). The results suggested that CAS alone could disrupt bacterial OM integrity. Consistently, checkerboard assays demonstrated that Mg^2+^ and EDTA resulted in a lower and higher synergistic degree of CAS in combination with rifampin against *E. coli* MG1655, respectively (Supplementary Figures 1A, 2B). Similarly, it was also found that CAS alone increased the fluorescence intensities of PI-labeled bacterial cells of the two test strains in a dose-dependent manner (Figures 3C,D). Additionally, the dose-dependent increased releases of β-galactosidase of the two test strains were also observed in the presence of CAS alone (Figures 3E,F). Notably, we also determined the bacterial membrane integrities and extracellular β-galactosidase releases in the presence of the combination of CAS and rifampin; however, there were no higher fluorescence intensities of NPN or PI-labeled bacterial cells of *E. coli* MG1655 and *E. coli* 72 treated with the combination of CAS and rifampin compared with CAS alone (Supplementary Figures 2A–D), and the β-galactosidase releases of the combination treatments were not higher than those of CAS acting alone (Supplementary Figures 2E,F). Taken together, these data hinted that CAS-induced bacterial envelope perturbation was a prerequisite for exhibiting the synergy with rifampin.

### 3.4 CAS changed the proton motivative force (PMF) of *Escherichia coli*

**Figure 4:**
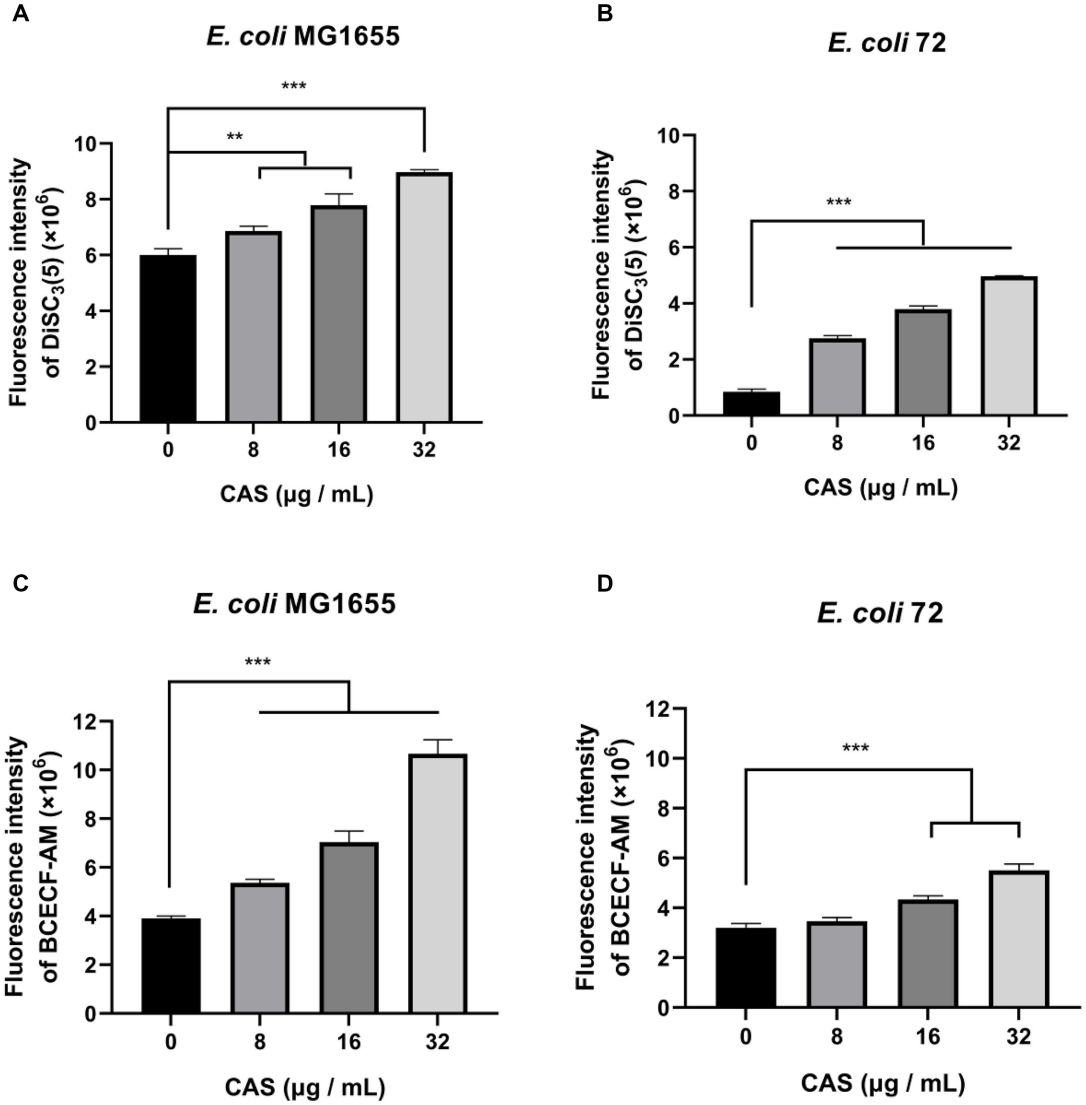
CAS dissipates the PMF of *E. coli*. The membrane potential-sensitive dye DiSC_3_(5) probed **(A)** *E. coli* MG1655 and **(B)** *E. coli* 72 were incubated with CAS (a final concentration of 8, 16, and 32 μg/mL) for 30 min, respectively. Next, the membrane potentials of samples were measured using an excitation wavelength at 622 nm and an emission wavelength at 670 nm with a microplate reader. The pH-sensitive BCECF-AM probed **(C)** *E. coli* MG1655 and **(D)** *E. coli* 72 were incubated with CAS (a final concentration of 8, 16, and 32 μg/mL) for 30 min, respectively. Next, the fluorescence intensities of samples were immediately monitored with an excitation wavelength at 488 nm and an emission wavelength at 535 nm. The data were analyzed by a two-tailed Student’s *t*-test in GraphPad Prism 7 software, with a value of **p < 0.01, ***p < 0.001. The experiments were carried out two times independently under the same conditions.

The PMF is an electrochemical gradient of protons across the cell membrane which is required for various bacterial cellular processes (Yang et al., 2023). PMF consists of the electric potential (ΔΨ) and the transmembrane proton gradient (ΔpH) (Yang et al., 2023). In this study, the membrane potential-sensitive dye DiSC_3_(5) was used to measure ΔΨ, and CAS led to an increase in fluorescence (Figures 4A,B), which implied that ΔΨ of *E. coli* MG1655 and *E. coli* 72 was dissipated. Additionally, the pH-sensitive probe BCECF-AM, was also used to measure intracellular pH, and CAS caused increased fluorescence in a dose-dependent manner in *E. coli* MG1655 and *E. coli* 72 (Figures 4C,D), which indicated that ΔpH value increased by alkalization of cytoplasm in *E. coli*. It could be seen that CAS disrupted the PMF of *E. coli*.

### 3.5 CAS inhibited biofilm formation of *Escherichia coli* by targeting biosynthesis of poly-GlcNAc mediated by PgaCD

**Figure 5:**
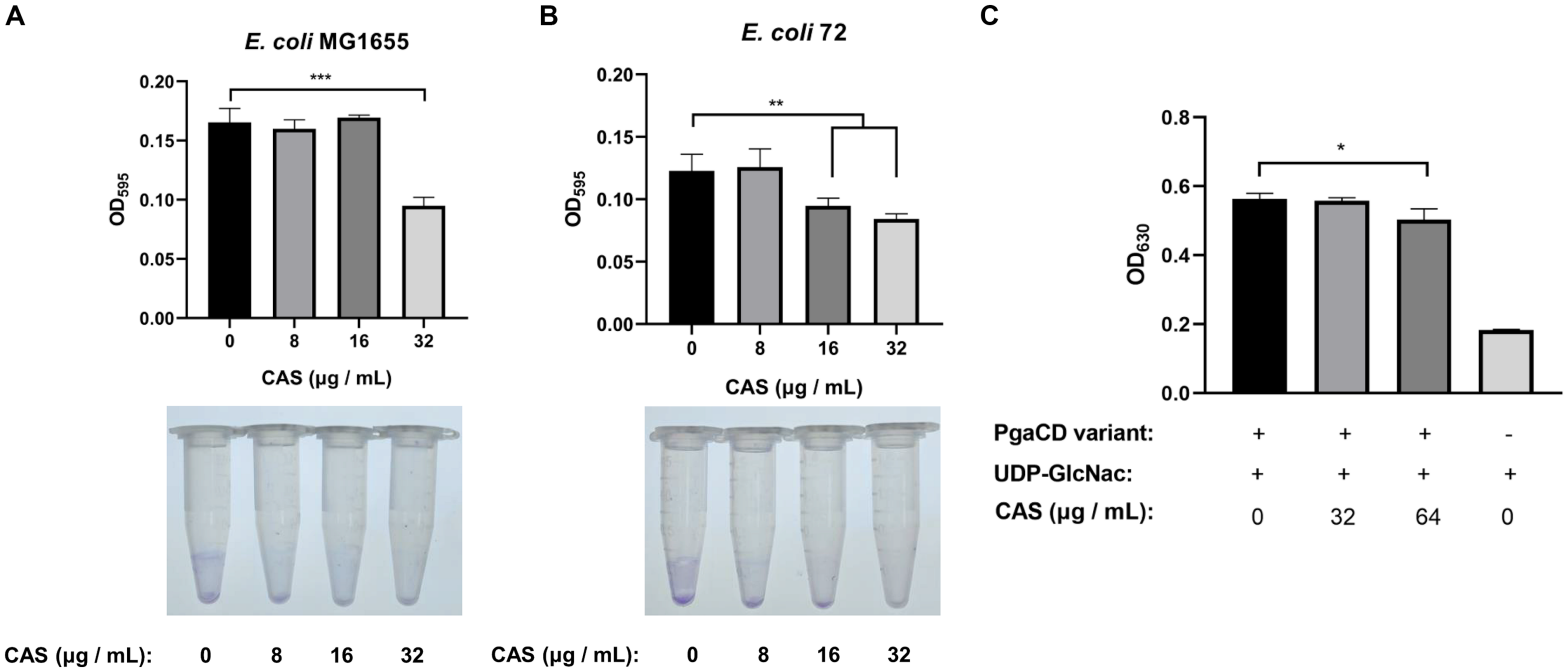
CAS inhibits the biofilm formation of *E. coli*. **(A,B)** Cells of *E. coli* MG1655 and *E. coli* 72 were grown to the OD_600_ of 0.1, followed by the addition of CAS (final concentrations of 8, 16, and 32 μg/mL), respectively. Subsequently, the bacterial cells were cultured for 48 h at 26°C, then strained using crystal violet to quantify the biofilm formation (top panel: biofilm formation in polystyrene 96-well microplate; bottom panel: visual presentation of biofilm in 1.5 mL polystyrene microtubes). **(C)** The enzymatic activity assay of PgaCD. Reaction mixture containing 0.3 mg/mL PgaC^V227L^PgaD^N75D/K76E^ variant, 5 mM MgCl_2_, 2 mM UDP-GlcNAc, and CAS (final concentrations of 32 and 64 μg/mL) or identical volume DMSO. Then, the mixtures were reacted for 18 h at 37°C, followed by centrifugation at 12,000 rpm at 4°C for 5 min. The UDP in the supernatants was indirectly measured using molybdate and malachite green to determine the enzymatic activity of the PgaCD variant. The data were analyzed by a two-tailed Student’s *t*-test in GraphPad Prism 7 software, with a value of *p < 0.05, **p < 0.01, ***p < 0.001. The experiments were carried out two times independently under the same conditions.

Biofilm is one of the intrinsic factors of AMR. It was reported in the literature that CAS could inhibit the biofilm formations of *S. aureus* by targeting IcaA, a synthase of poly-N-acetylglucosamine polymers, sharing homology with fungal β-1-3-glucan synthase (a pharmacological target of CAS) (Siala et al., 2016). Consistently, our study also showed that CAS alone inhibited the biofilm formations of *E. coli* MG1655 and *E. coli* 72 strains (Figures 5A,B). However, we found that the cultures of *E. coli* 72 rather than *E. coli* MG1655 treated with the combination of CAS and rifampin formed more biofilm compared with CAS alone, but still significantly less biofilm compared with blank treatment (Supplementary Figures 3A,B). Furthermore, by bioinformatics, PgaC in *E. coli* was deemed as a homologous protein to IcaA. Previous studies showed that the enzymatic activity of the PgaC^V227L^PgaD^N75D/K76E^ variant is independent of the regulation of c-di-GMP (Steiner et al., 2013). The variant complex was therefore expressed and purified for an enzymatic assay (Supplementary Figure 4). The results showed that CAS indeed inhibited the biosynthesis of poly-GlcNAc mediated by the variant of PgaCD (Figure 5C). Taken together, a tentative conclusion can be drawn that CAS inhibited biofilm formation of *E. coli* by disturbing biosynthesis of poly-GlcNAc mediated by PgaCD.

### 3.6 Transcriptome analysis of *Escherichia coli* MG1655 treated with CAS

**Figure 6:**
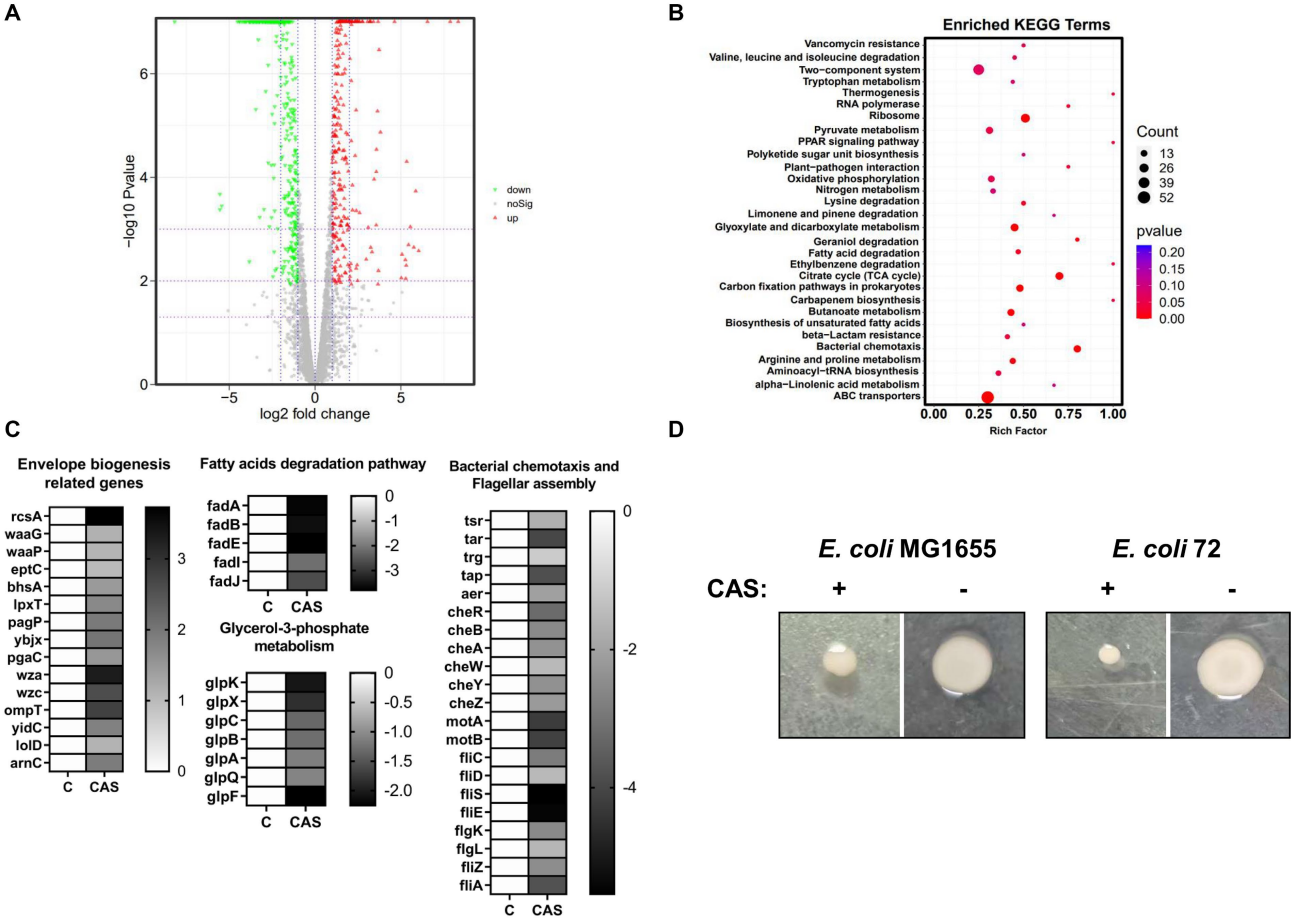
The transcriptomic analysis. **(A)** The volcano plot of DEGs; **(B)** The enriched KEGG analysis; **(C)** The DEGs associated with envelope biogenesis, fatty acid degradation, glycerol-3-phospholate metabolism, bacterial chemotaxis, and flagellar assembly were listed in heatmaps; **(D)** The swarming mobility of *E. coli* MG1655 and *E. coli* 72 treated with or without CAS (64 μg/mL), respectively.

To further explore the antimicrobial mechanisms of CAS, a transcriptome analysis of *E. coli* MG1655 treated with CAS was performed. A total of 606 differently expressed genes (DEGs) were identified between the test group (*E. coli* MG1655 treated with CAS) and the control group (Figure 6A; Supplementary Table 6). Subsequently, the reliability of transcriptome data was further confirmed by quantitative RT-PCR (Supplementary Figure 5). KEGG enrichment analysis revealed that these DEGs in the test group were involved in bacterial chemotaxis, citrate cycle, ABC transporters, quorum sensing, and metabolism-related pathways (Figure 6B). Notably, a part of DEGs required for bacterial envelope stress responses and membrane biogenesis were upregulated in the test group, such as *rcsA*, *waaG*, and *waaP*. Conversely, *fadA*, *fadB*, *fadE*, *fadI*, and *fadJ*, which are involved in the fatty acid (the building blocks of the plasma membrane) degradation, were downregulated (Figure 6C). These changes in gene expression profile were consistent with *E. coli* treated with polymyxin targeting the bacterial membrane (Nang et al., 2022). Altogether, these data hinted that the membrane biogenesis pathways of *E. coli* treated with CAS were activated as feedback. Additionally, the genes related to glycerol-3-phosphate metabolism were significantly downregulated in the test group (Figure 6C), which also agreed with previous studies that the decreased levels of glycerol-3-phosphate-conferred antibiotic tolerance (Kurabayashi et al., 2015). Interestingly, bacterial chemotaxis and flagellar assembly are other important enriched pathways in DEGs (Figure 6C). To verify the consistency between these gene-expressed profiles and phenotypes, we performed a bacterial swarming mobility assay. The results showed that CAS at 64 μg/mL dramatically inhibited the swarming mobility of *E. coli* MG1655 and *E. coli* 72 (Figure 6D). Bacterial mobility is a prerequisite for bacterial biofilm formation. This may also be a mechanism of CAS-mediated perturbation of bacterial biofilm formation. In summary, the transcriptome data support these phenotypes that CAS destroyed bacterial envelope integrity and inhibited bacterial motility.

### 3.7 The bioactive evaluations of analoges of CAS

**Figure 7:**
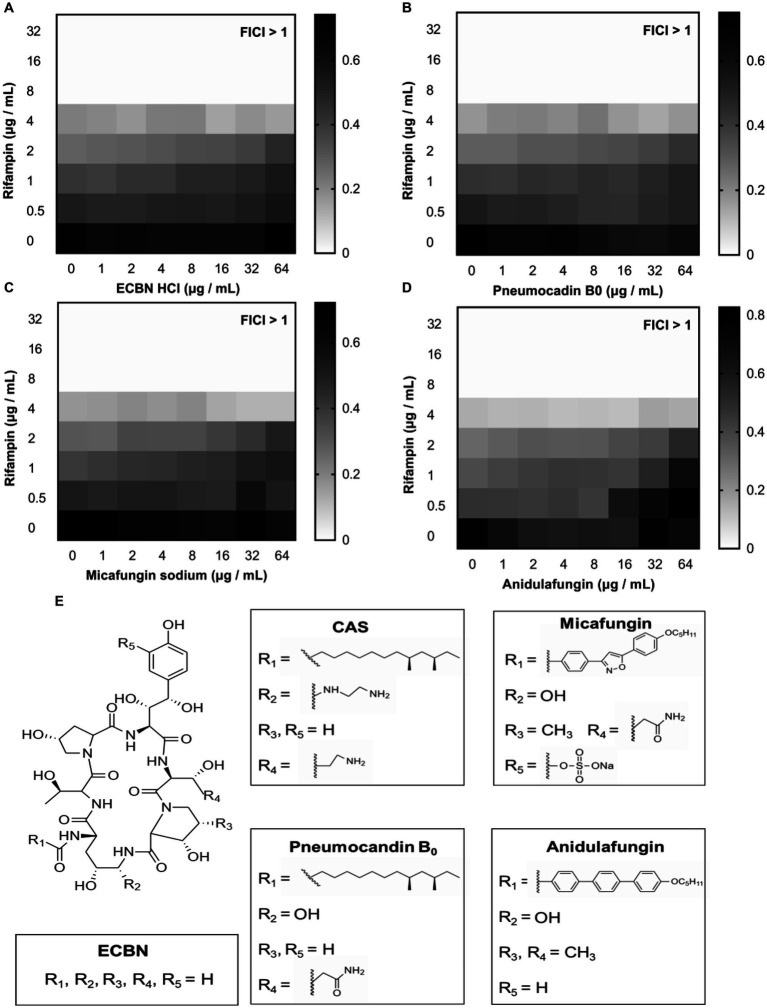
The analogs of CAS. The synergistic effect of **(A)** ECBN HCl, **(B)** Pneumocandin B_0_, **(C)** Micafungin sodium, and **(D)** Anidulafungin with rifampin against *E. coli* MG1655, respectively. The grayscale values of the 8 × 8 checkerboard represent the OD_600_ values; an FIC index of <0.5 is used to define synergy. **(E)** The structures of ECBN, CAS, Pneumocandin B_0_, Micafungin, and Anidulafungin.

To find out the higher bioactive antibiotic adjuvants and figure out the structure–activity relationship (SAR) of CAS, the echinocandin B nucleus and its three derivatives were subjected to determine its synergistic degree with rifampin against *E. coli* MG1655. Unfortunately, checkerboard assays suggested that all compounds, including Micafungin sodium, Anidulafungin, Pneumocandin B_0_ and Echinocandin B nucleus hydrochloride (ECBN HCL), displayed a weak synergistic activity with rifampin (Figures 7A–D). Subsequently, the structural difference of these compounds was analyzed. It was shown in Figure 7E that CAS was obtained by replacing the negatively charged hydroxyl group and R group of 3-hydroxyglutamine of Pneumocandin B_0_ with a cationic aminoethyl ether group and the R group of 3-hydroxyornithine, respectively, which indicated that the positively charged moieties of CAS may increase its affinity with phospholipids with negative charges, hence, enhance its potency. Additionally, hydrophobic fatty acid chain of CAS attached to the echinocandin B nucleus has been considered an important moiety for its attachment to fungal membranes and antifungal potency (Szymański et al., 2022). Consistently, it is most likely to play a role in attaching the cell membrane of GNB.

### 3.8 Resistance development study

**Figure 8:**
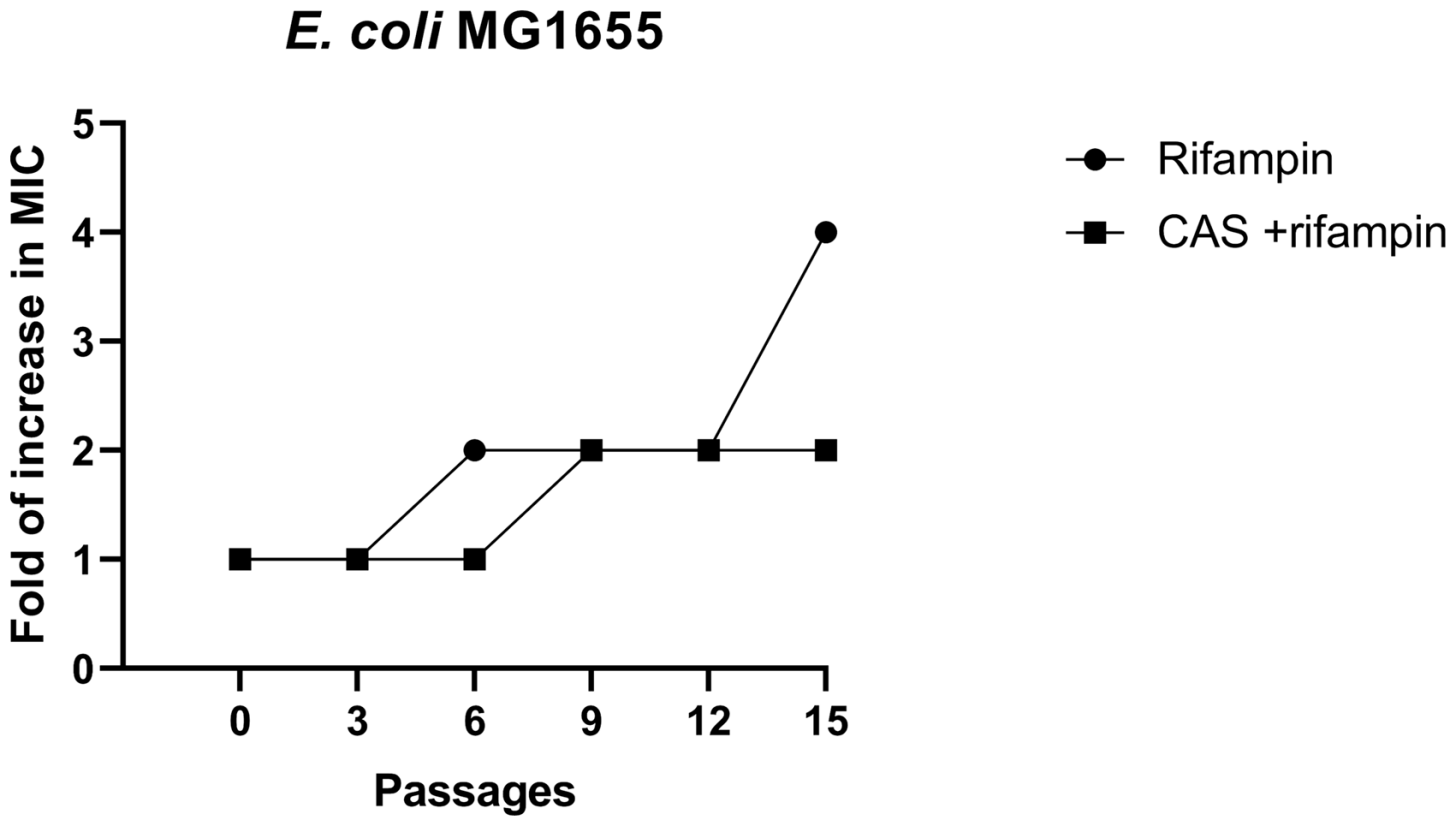
Resistance development. A comparison of fold increase in MIC of rifampin (4 μg/mL) and combined with CAS (8 μg/mL) against *E. coli* MG1655.

The rapid antibiotics resistance development of *E. coli* is alarming. In this study, the rifampin resistance development of drug-sensitive *E. coli* MG1655 in the presence of CAS was performed. The results showed that a 2- to 4-foldchange increase in MIC values of rifampin against *E. coli* MG1655 treated with rifampin alone or combined with CAS was observed (Figure 8). Hence, CAS was less susceptible to accelerating the resistance development of *E. coli* MG1655.

### 3.9 The synergistic efficacy of CAS in combination with rifampin against AMR *Escherichia coli* strain *in vivo*

**Figure 9:**
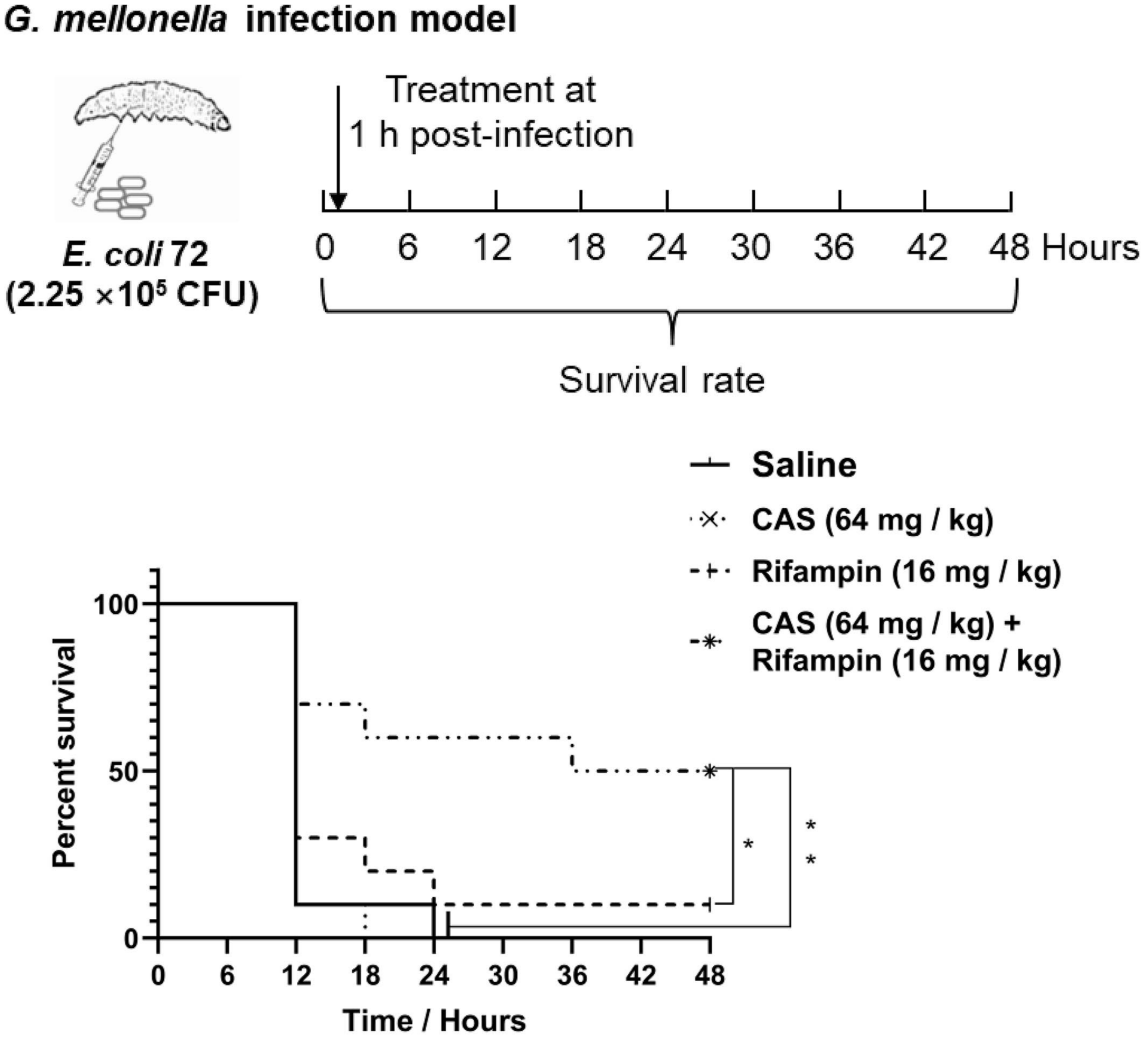
*Galleria mellonella* larvae infection assay. *G. mellonella* larvae were inoculated with CAS at 64 mg/kg × body weight and rifampin at 16 mg/kg × body weight alone or in combination after *E. coli* 72 infection. The survival was recorded every 6 h for 48 h. The survival data were analyzed by log-rank test in GraphPad Prism 7 software, with a value of *p < 0.05, **p < 0.01. The experiments were carried out two times independently under the same conditions.

To further assess the potency of CAS in combination with rifampin *in vivo*, we utilized the *G. mellonella* larvae infection model for evaluating the virulence of multidrug-resistant strain *E. coli* 72. It was shown that CAS at 64 mg/kg body weight and rifampin at 16 mg/kg body weight alone could not reduce the death rate of *G. mellonella* larvae challenged with *E. coli* 72; however, a combination of the two drugs significantly reduced the mortality rate of *G. mellonella* larvae challenged with *E. coli* 72 (Figure 9). In summary, CAS also potentiated the efficacy of rifampin *in vivo*.

## 4 Discussion

AMR has become a global health concern, resulting in huge economic and life losses, in particular for AMR of Gram-negative pathogens (Mukerji et al., 2017; Dettori et al., 2023). To date, developing novel antimicrobial strategies is still one of the most effective strategies to combat AMR. Recently, antibiotic adjuvants have been deemed as an attractive approach to extending the life span of existing antibiotics (Sharma et al., 2021). In this study, CAS, an antifungal agent, was identified as an antibiotic adjuvant that has low antimicrobial activity against GNB by itself but could potentiate the antimicrobial efficacy of rifampin or colistin against GNB strains *in vitro* or *in vivo*. Therefore, the mechanism that results in the efficacy of CAS as an antibiotic adjuvant is curious. Our data demonstrated that the modes of action of CAS included destroying envelope integrity, dissipating PMF, and inhibiting biofilm formation.

CAS is a large molecule of more than 1,000 Da, while the cutoff for the compound to permeate the outer membrane is approximately 600 Da (O'Shea and Moser, 2008). We therefore considered that CAS directly acted on bacterial membrane partition. Previous studies revealed that the hydrophobic fatty acid chain attached to the echinocandin B core of CAS is vital for its antifungal activity because it acts as a “hook” that allows the drug to anchor in the fungal cell membrane; similarly, the hydrophobic fatty acid tail of CAS probably facilitated its bound to bacterial membrane partition (Szymański et al., 2022). Additionally, the different antimicrobial activity between CAS and Pneumocandin B_0_ indicated that the cationic moieties of CAS are also beneficial to its higher efficacy, which was possibly due to its higher affinity with bacterial membrane partition by being tightly bound to negatively charged LPS and phospholipid. The phenotype was also supported by the results that the addition of Mg^2+^ in the MHB medium could counteract the bioactivity of CAS, while EDTA could enhance its bioactivity. Taken together, it was tentatively concluded that the cationic moieties and hydrophobic fatty acid tail of CAS are required for its binding to bacterial membrane partition. Recent studies have found that the antimicrobial activity of rifampin against GNB could be potentiated by several compounds disrupting OM integrity, such as SLAP-S25, PMBN, and Peptide hLF1-11 (Vaara, 1992; Song et al., 2020; Morici et al., 2023). Consistently, our data also showed CAS could impair bacterial outer and inner membrane integrities and upregulate the expression of genes associated with bacterial membrane biogenesis. Therefore, it is most likely that the binding of CAS to the bacterial membrane leads to disruption and loss of bacterial envelope integrity, causing the entry of antibiotics.

PMF is utilized as the energy source for material transport, flagellar motility, ATP synthesis, and multidrug efflux pump (Paul et al., 2008). PMF is composed of ΔΨ and ΔpH, which was associated with antibiotic uptake (Mitchell, 1961; Morici et al., 2023). Previous studies showed that tetracycline uptake is driven by ΔpH (Yamaguchi et al., 1991), whereas aminoglycoside uptake depends on ΔΨ (Taber et al., 1987). In this study, we found that PMF was also a potential target of CAS. CAS dissipated ΔΨ, and then the ΔpH compensatively increased. PMF disruption is associated with bacterial viability and bacterial susceptibility to antibiotics, leading to it becoming an attractive target of antibiotics or adjuvants (Yang et al., 2023). For example, daptomycin, HT61, and telavancin dissipate bacterial ΔΨ, further resulting in permeabilizing and depolarizing the cytoplasmic membrane (Hubbard et al., 2017; Ma et al., 2017; Al Jalali and Zeitlinger, 2018). In addition, both 9-aminoacridine and pixantrone resensitized Gram-negative pathogens to rifampin by disrupting its PMF (She et al., 2022, 2023). Hence, PMF disruption is one of the mechanisms of CAS as a rifampin enhancer.

Biofilms are surface-associated bacterial communities that cause chronic and persistent infections (Costerton et al., 1999; Aswathanarayan et al., 2023). Bacteria that produce biofilms are more resistant to antibiotics (Hall and Mah, 2017). Therefore, anti-biofilm is an effective antimicrobial strategy (Ong et al., 2018). Several anti-biofilm compounds indeed recovered bacterial susceptibility to antibiotics; for example, aspartic acid and glutamic acid could potentiate the antimicrobial activity of ciprofloxacin against *S. aureus* by combating biofilm (Warraich et al., 2020), and the anti-biofilm agent PgTeL showed synergy with ceftazidime against resistant *E. coli* isolates (da Silva et al., 2019). Our data also showed that CAS inhibited the biofilm formation of *E. coli*. It is well-established that multiple factors can influence bacterial biofilm formation, including bacterial motility (Prüß, 2017). Bacterial motility is the first phase of biofilm development, which positively correlates with bacterial biofilm thickness in *E. coli* (Wood et al., 2006). We also found that CAS suppressed the swarming motility of *E. coli*, and transcriptome results also showed that bacterial chemotaxis and flagellar assembly-related genes were significantly downregulated. These results probably concluded that bacterial biofilm formation inhibition by CAS is likely through influencing bacterial motility. In addition, there are possibilities that other mechanisms could also explain the anti-biofilm bioactivity of CAS. CAS is commonly used as an antifungal drug of a class of echinocandin, which targets β-1-3-glucan synthetase of fungi (Hu et al., 2023). Recently, CAS has also been discovered to inhibit the enzymatic activity of IcaA (the homolog protein of β-1-3-glucan synthetase of fungi) required for the biosynthesis of poly-N-acetylglucosamine polymers of *S. aureus*, further preventing its biofilm formation (Siala et al., 2016). Moreover, CAS could increase fluoroquinolone penetration inside biofilms, leading to its higher antimicrobial activity *in vitro* or *in vivo* (Siala et al., 2016). Similarly, this study found that CAS was also dependent on its inhibition of PgaC of *E. coli* sharing homology with IcaA and resulted in bacterial biofilm formation deficiency. Most importantly, CAS disrupted the biofilm formation of *E. coli* and did not accelerate bacterial resistance development, which hints that the combination of CAS and rifampin can be used for the long term.

CAS, as an FDA-approved drug, possesses favorable pharmacokinetic properties and an excellent safety profile (Groll and Walsh, 2001). Nevertheless, one major limitation of CAS is the lack of an oral formulation (Morrison, 2005), and further modification or novel dosage form research on CAS may contribute to its oral administration.

## 5 Conclusion

In summary, an FDA-approved antifungal agent, CAS, was identified to show a synergy effect with rifampin or colistin against not only sensitive but also resistant GNB strains, which implies that the mode of action of CAS may not be limited to targeting resistant mechanisms. Indeed, we found that the mechanisms of action of CAS are mainly involved in destroying envelope integrity, dissipating PMF, and inhibiting biofilm formation. Collectively, CAS may have the potential for future treatment of multidrug-resistant infections.

## Data Availability

The datasets presented in this study can be found in online repositories. The names of the repository/repositories and accession number(s) can be found in the article/Supplementary material.

## Footnotes

^1^ http://www.genome.jp/kegg/
