# Supplementary material for: Caspofungin enhances the potency of rifampin against Gram-negative bacteria
Source: Front Microbiol. 2024 Aug 15;15:1447485. doi: 10.3389/fmicb.2024.1447485 (PMC11358092; doi:10.3389/fmicb.2024.1447485)
Supplement: Supplementary file 1 [file Data_Sheet_1.docx]

Supplementary Material

**Supplementary Table 1. Bacterial strains and plasmid used in this study.**

| Strains or Plasmids | Description | Reference or source |  |
| --- | --- | --- | --- |
| *E. coli* MG1655 | Laboratory strain | Laboratory stock |  |
| *E. coli* ATCC25922 | *E. coli* standard strain | Laboratory stock |  |
| *E. coli* 69 | Antimicrobial-resistant *E. coli* strain | Laboratory stock |  |
| *E. coli* 72 | Multiple drug-resistant *E. coli* strain | Laboratory stock |  |
| *S.* Typhimurium ATCC14028 | *S.* Typhimurium standard strain | Laboratory stock |  |
| *P. aeruginosa* PAO1 | | *P. aeruginosa* standard strain | Laboratory stock |
| *P. aeruginosa* 2619 | Antimicrobial-resistant *P. aeruginosa* strain | Laboratory stock |  |
| pTrc99a::*pgaC*^V227L^*pgaD*^N75D/K76E^ | The recombinant plasmid for the expression of PgaCD variant of *E. coli* | This study |  |

**Supplementary Table 3. Primers used in this study.**

| Primers | Sequences (5’-3’) | Description |
| --- | --- | --- |
| motA-1 | TGGAAGCCTTGGAGCACTCTATC | qRT-PCR for *motA* |
| motA-2 | TGGTGTATTTGGAGCGACGAAAC |  |
| motB-1 | ACTGGCGACTGCGGTTACG | qRT-PCR for *motB* |
| motB-2 | ATGTTCGGCTGCTTATTCACTTCC |  |
| yjcH-1 | CGCCGCTGAATCCGAACAC | qRT-PCR for *yjcH* |
| yjcH-2 | TCGCCCGCCAGATGTAGATAC |  |
| fliA-1 | TACAGGCGGGCGGCATTG | qRT-PCR for *fliA* |
| fliA-2 | GTCACGGCTGCGAAGTTCATC |  |
| fadE-1 | CTGGCGGATGCTGGTGGAG | qRT-PCR for *fadE* |
| fadE-2 | TTGAACTGACGGCGAATGTGAG |  |
| ynfM-1 | GGCGTGGTCCAGTGATGTTG | qRT-PCR for *ynfM* |
| ynfM-2 | GCGAAGAATCCTGCTGAGAAGAG |  |
| dtpA-1 | ACTACCGTAACCTGCTGCTGAC | qRT-PCR for *dtpA* |
| dtpA-2 | CCACGATACCGAAGGCAACAAC |  |
| cadA-1 | CCACCTGATGATGATGAGCGATG | qRT-PCR for *cadA* |
| cadA-2 | TGGAATTCACTCTGTGGGATACCAC |  |
| treC-1 | GTGGTGCTATCGCCGTGAATG | qRT-PCR for *treC* |
| treC-2 | GGTGAGGCTTCTTCGTAGTTATGC |  |
| ompT-1 | ACAGCCAGAGGTGGTTCCTATATC | qRT-PCR for *ompT* |
| ompT-2 | ACGATAACTTCCAGTCAAGCCAATG |  |
| gadph-1 | CGCTTCCCAGAACATCATCCC | qRT-PCR for gadph |
| gadph-2 | GAACGGTCAGGTCAACTACAGATAC |  |
| pgaCD-1 | CACACAGGAAACAGACCATGGAAATGATTAATCGCATCGTATC | The first fragment of *pgaCD* variant coding sequence |
| pgaCD-2 | TAACACCGGAAACGGTAAATAAGTTTCCATAGATACGCTG |  |
| pgaCD-3 | CAGCGTATCTATGGAAACTTATTTACCGTTTCCGGTGTTA | The second fragment of *pgaCD* variant coding sequence |
| pgaCD-4 | GTTTTTGAAAACGCAGCTCATCGTACAGCGCCCAGACAAT |  |
| pgaCD-5 | ATTGTCTGGGCGCTGTACGATGAGCTGCGTTTTCAAAAAC | The third fragment of *pgaCD* variant coding sequence |
| pgaCD-6 | TCCGCCAAAACAGCCAAGCTTTCATTTTTCGAACTGCGGGTGGCTCCATGCCCGGACTAGCGCTTTTT |  |

**Supplementary Table 5. FICI values of CAS in combination with rifampin or colistin against *E. coli*, *S.* Typhimurium, *P. aeruginosa*.**

|  | **CAS** | |
| --- | --- | --- |
| **Strains** | **Rifampin** | **Colistin** |
| *E. coli* MG1655 | ≤0.25 | ≤0.15625 |
| *E. coli* ATCC25922 | ≤0.1875 | ≤0.1328125 |
| *E. coli* 69 | ≤0.15625 | ≤0.2578125 |
| *E. coli* 72 | ≤0.15625 | >0.5 |
| *S.* Typhimurium ATCC14028 | ≤0.5 | ≤0.25 |
| *P. aeruginosa* PAO1 | ≤0.375 | ≤0.5 |
| *P. aeruginosa* 2619 | ≤0.375 | ≤0.2578125 |

**
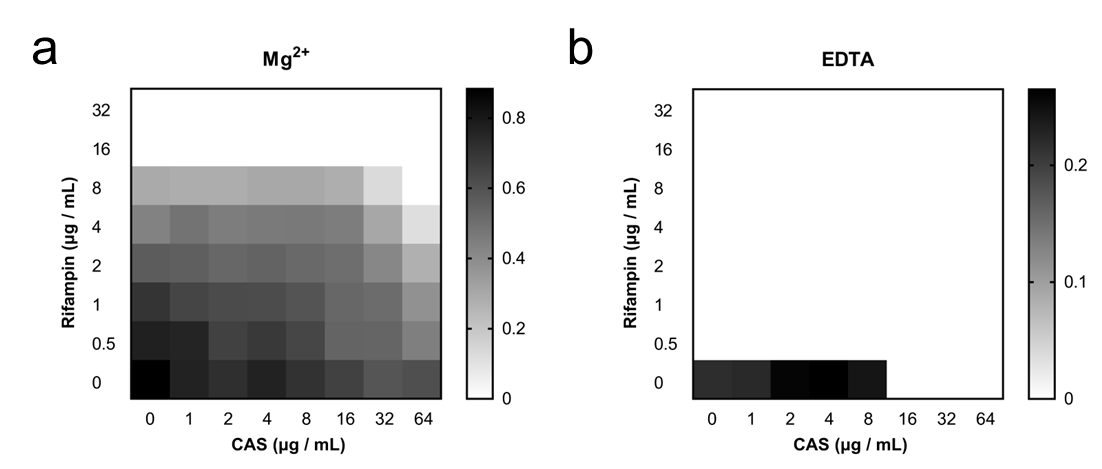
**

**Supplementary Figure 1. Checkboard assay.** The effects of (a) Mg^2+^ (10 m M) and (b) EDTA (1 mM) on the synergistic degree of CAS in combination with rifampin against *E. coli* MG1655 strain, respectively.


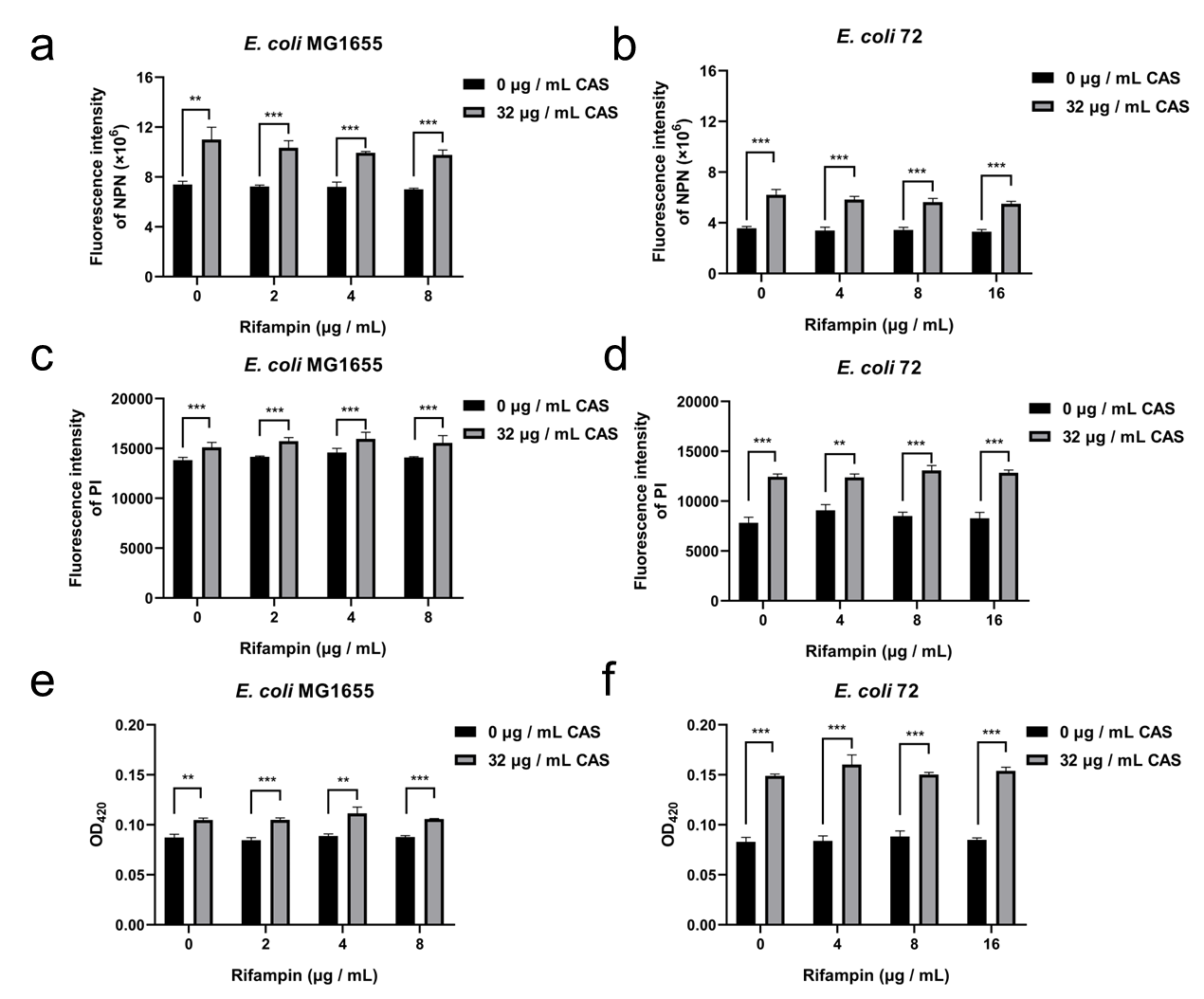


**Supplementary Figure 2.** **The effect of the combination of CAS and rifampin on the bacterial envelope of *E. coli*.** The dye NPN (a final concentration of 10 μM) probed (a) *E. coli* MG1655 and (b) *E. coli* 72 were incubated with rifampin or combined with CAS for 30 min, respectively. Next, fluorescence was measured on a microplate reader with the excitation wavelength at 350 nm and the emission wavelength at 420 nm. The PI (a final concentration of 10 nM) was added to the cells of (c) *E. coli* MG1655 and (d) *E. coli* 72 in the presence of rifampin or combined with CAS, respectively. After incubation for 30 min, fluorescence was measured with an excitation wavelength at 535 nm and an emission wavelength at 615 nm. The cells of (e) *E. coli* MG1655 and (f) *E. coli* 72 treated with rifampin or combined with CAS were centrifuged, and the supernatants were incubated with a final concentration of 3 mM ONPG for 30 min. The absorbance at 420 nm was measured using a microplate reader. The data were analyzed by a two-tailed Student’s t-test in GraphPad Prism 7 software, with a value of p < 0.05, *; p < 0.01, **; p < 0.001, ***.


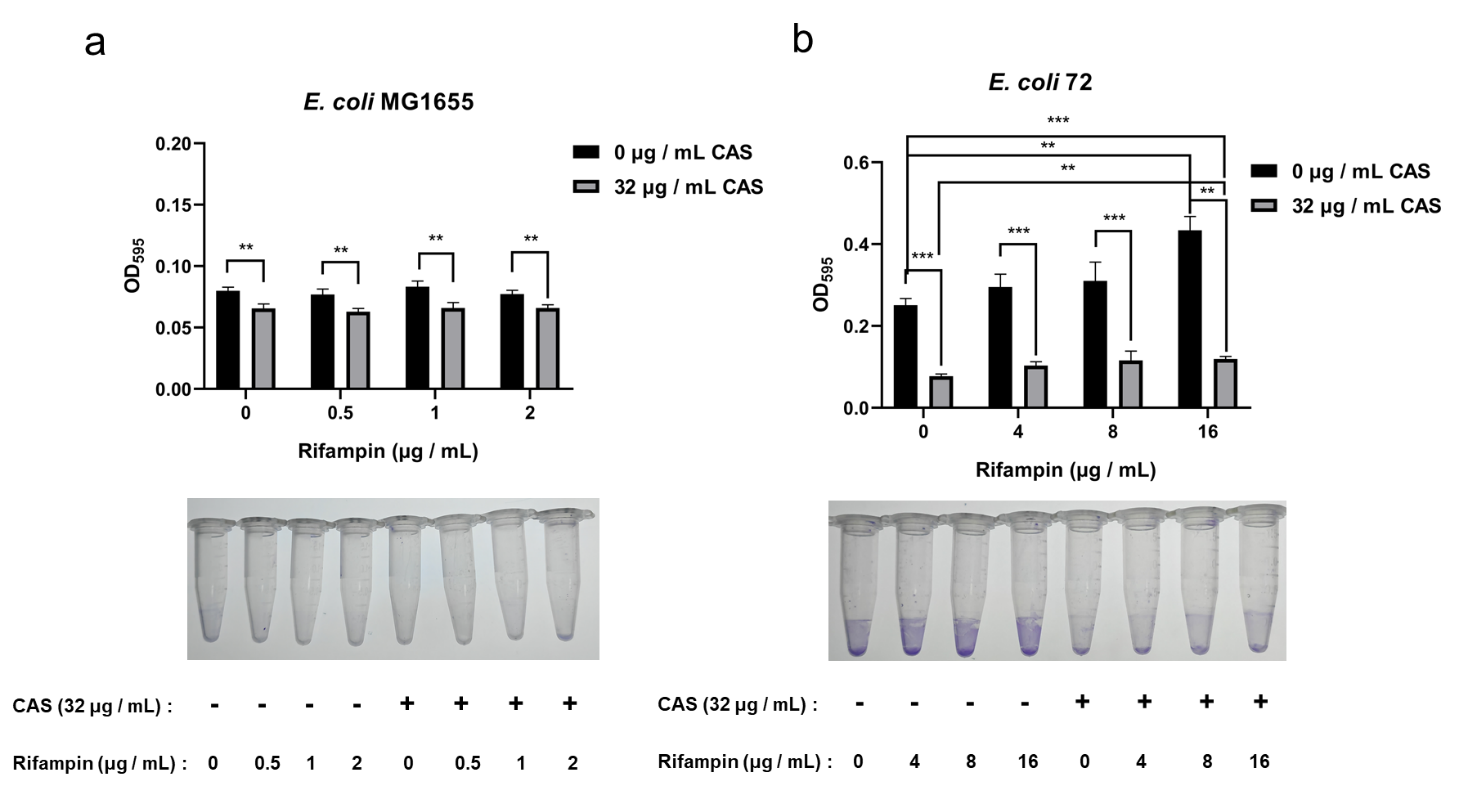


**Supplementary Figure 3. The effect of the combination of CAS and rifampin on bacterial biofilm formation of *E. coli*.** (a) and (b) Cells of *E. coli* MG1655 and *E. coli* 72 were grown to the OD_600_ of 0.1 in LB medium containing 0.2% glucose, followed by the addition of rifampin or combined with CAS, respectively. Subsequently, the bacterial cells were cultured for 48 h at 26 ℃, then, were strained using crystal violet to quantify the biofilm formation (Top panel: biofilm formation in polystyrene 96-well microplate, Bottom panel: visual presentation of biofilm in 1.5 mL polystyrene microtubes).


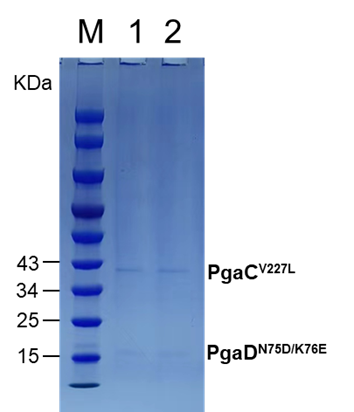


**Supplementary Figure 4. The SDS-PAGE analysis of the purified PgaCD variant.**


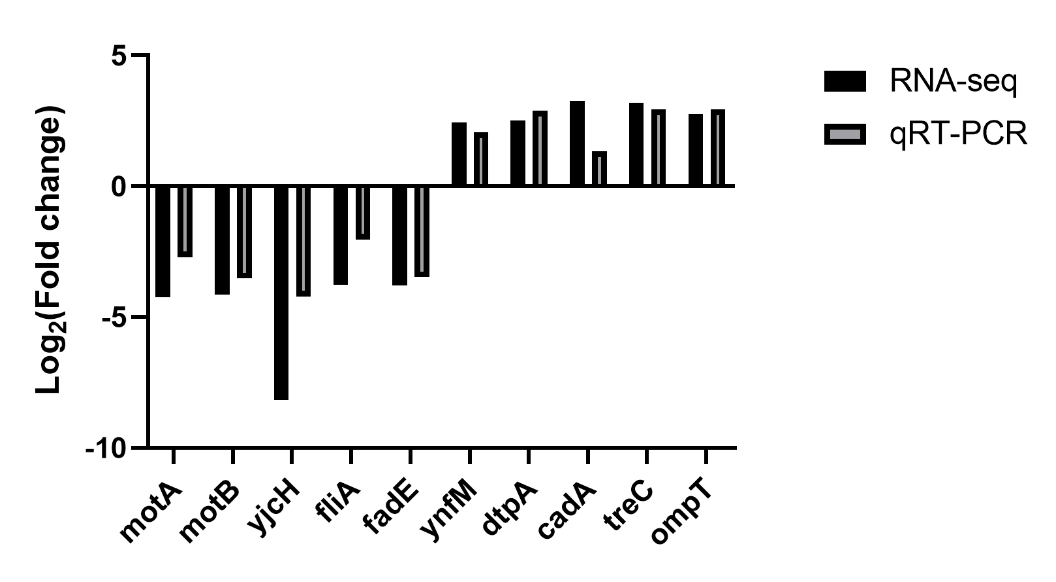


**Supplementary Figure 5.** **The verifications of the transcriptome using qRT-PCR.** Relative gene expression levels of 10 genes (*motA*, *motB*, *yjcH*, *fliA*, *fadE*, *ynfM*, *dtpA*, *cadA*, *treC*, *ompT*) in *E. coli* MG1655 treated with CAS were detected using quantitative RT-PCR.
